# The association between mindfulness, resilience, and academic achievement of pharmacy students in Saudi Arabia

**DOI:** 10.3389/fpubh.2024.1446460

**Published:** 2024-10-22

**Authors:** Mona Almanasef, Dalia Almaghaslah

**Affiliations:** Department of Clinical Pharmacy, College of Pharmacy, King Khalid University, Abha, Saudi Arabia

**Keywords:** mindfulness, resilience, academic performance, pharmacy students, Saudi Arabia

## Abstract

**Background:**

Literature suggests that mindfulness and resilience positively impact academic performance. This study was conducted to assess mindfulness and resilience levels among pharmacy students. The study also aimed to explore the relationship between mindfulness and resilience, as well as their effects on GPA, which serves as an indicator of academic performance.

**Methods:**

The study utilized the Five Facet Mindfulness Questionnaire (FFMQ), a validated tool consisting of 39 items across five domains: observing, describing, acting with awareness, non-judging of inner experience, and non-reactivity. Additionally, the Brief Resilience Scale (BRS), a validated questionnaire with 6 items using a 5-point Likert scale, was employed to assess resilience.

**Results:**

The average scores obtained from the mindfulness and resilience scales were 3.00 (SD = 0.36) and 3.00 (SD = 0.65), indicating intermediate levels of resilience and mindfulness, respectively. No statistically significant differences were found in mindfulness scores between male and female students, or between students with and without a previous diagnosis of mental health issues. However, a statistically significant higher level of resilience was observed among students who have never been diagnosed with mental health issues compared to those who reported a previous diagnosis. However, females scored significantly higher in the observing domain of mindfulness compared to males, while males scored significantly higher in the acting with awareness domain. Students who had never been diagnosed with mental health issues scored significantly higher in acting with awareness. A significant positive association was found between resilience and mindfulness scores of the students (r = 0.45, *p* < 0.001). However, significant positive correlation was found between resilience and describing as well as acting with awareness mindfulness, *p* < 0.05. Assessing the association between student GPA and mindfulness as well as resilience scores showed a weak association.

**Discussion and conclusion:**

Tailored interventions and programs could be implemented to foster resilience, enhance students’ ability to cope, and equip them with tools to bounce back in the face of adversity. Further research could explore other factors that might influence the strength of the relationships between mindfulness and resilience, and student GPA.

## Introduction

University life, especially in demanding programs such as pharmacy, often presents considerable stressors that can affect students’ mental health and their academic performance. The Transactional Model of Stress and Coping emphasizes that stressors—events or situations perceived as challenging—can overwhelm an individual’s capacity to cope when their perceived resources are insufficient (1, 2).

Research indicates that college can be a period of significant psychological distress and anxiety for some students (3, 4). Stress often manifests as anxiety and depression (3, 4). Literature suggests that pharmacy students experience heightened stress levels, which can lead to a diminished quality of life (5). Stress is known to negatively impact attention and concentration, and the lack of effective coping skills can hinder a student’s ability to learn effectively in the classroom (6).

The rigorous demands of the pharmacy curriculum, frequent exams, accumulating responsibilities, competition for postgraduate placements, preparation for licensure exams, and a competitive environment contribute to higher stress levels among pharmacy students compared to the general population, regardless of their year of study (7, 8). Additionally, stress experienced by student pharmacists is associated with ongoing stress in their professional practice, with pharmacists being among the healthcare professionals with the highest levels of burnout (9–11).

Mindfulness is defined as “the capacity to focus attention on the present moment, both externally and internally” (3). Higher levels of mindfulness have been positively correlated with Grade Point Average (GPA) and academic performance (3). Kabat-Zinn’s Mindfulness-Based Stress Reduction (MBSR) is a well-regarded application of mindfulness meditation, characterized by deliberately paying attention to the present moment with intention and without judgment (12).

Several mindfulness self-assessment tools are available, including the Kentucky Inventory of Mindfulness Skills, Freiburg Mindfulness Inventory, Cognitive and Affective Mindfulness Scale, Revised Mindful Attention Awareness Scale, and Southampton Mindfulness Questionnaire (13, 14). The Five Facet Mindfulness Questionnaire (FFMQ) was developed based on these validated tools and includes 39 items across five facets: observe, describe, act with Awareness, nonjudge, and nonreact (13, 14). Observe refers to noticing sensory experiences—both from external sources and within the body—along with related thoughts and emotions. Describe involves labelling internal experiences with words to articulate them. Act with Awareness emphasizes maintaing continuous attention and awareness of present activities and experiences. The Characteristic of Nonjudge involves adopting a non-evaluative attitude towards one’s thoughts and emotions, focusing on inner experiences without criticism. Nonerect refers to perceiving thoughts and feelings, particularly distressing ones, without feeling compelled to react or being overwhelmed (15).

Research on mindfulness has revealed associations with various demographic factors, including age and gender (16). A cross-sectional study conducted in Bosnia using the FFMQ found that mindfulness tends to increase with age (16). This study support previous research which reported that older individuals generally exhibit greater emotional control, enhanced present-moment awareness, and reduced self- and other-judgment (16–19).

Gender differences in mindfulness were also noted (16). Females were found to score higher on the observing facet of mindfulness, indicating a greater tendency to notice and be aware of internal and external experiences (16). In contrast, males scored higher on the acting with awareness facet, suggesting they may be more focused on being present in their actions (16). Additionally, another study found that while females reported higher overall mindfulness levels, men showed a stronger association between mindfulness and happiness (19).

.Mindfulness and psychological well-being were found to be high determinants of resilience (20). Resilience, an abstract concept describing the ability to overcome or adapt to adverse circumstances, is also a crucial coping capability (21). Specifically, academic resilience refers to a student’s capacity to recover and make progress after setbacks such as failing an exam or course (21).

Two studies explored resilience among pharmacy students. The first study used the Connor-Davidson Resilience Scale-25 to investigate how elements of the MPharm curriculum contribute to resilience (22). It found that activities such as making mistakes in a safe environment and the need to achieve high grades were particularly valuable for resilience development. The second study, a cross-sectional analysis, measured academic resilience with the Academic Pharmacy Resilience Scale and evaluated academic success based on Pharmacy math course pass rates (23). However, it found no significant association between resilience scores and math course results.

Empirical evidence has evaluated the impact of various socio-contextual factors on resilience, including gender, age, ethnicity, socio-economic status, and environmental triggers. This research highlights how these variables interact to shape individuals’ ability to withstand and adapt to adversity (24–27). Research on the impact of gender on resilience revealed diverse findings. Sahar and Muzaffar (28) argue that females often demonstrate higher resilience due to their capacity for expressing positive emotions and maintaining supportive networks (28). In contrast, Naz et al. (27) suggest that males might exhibit greater resilience through internal strengths and personal coping mechanisms. These differing perspectives highlight the complex interplay between gender and resilience, underscoring the influence of emotional expression and social support for women, as well as internal resources for men. Such variations can be attributed to cultural and societal factors that shape how resilience is developed and expressed across genders (27).

Previous studies conducted at the same institution as the current study include a cross-sectional survey that evaluated mental health literacy and help-seeking behaviors using the Mental Health Literacy Scale (MHLS) and the General Help-Seeking Questionnaire (GHSQ). This study found that pharmacy students had lower levels of mental health literacy and were less likely to seek help. Mental health problems such as stress, depression, and anxiety were reported to have long-term negative effects on physical, social, and mental outcomes, as well as academic achievement (29).

Despite the growing body of research, studies focusing on mindfulness and resilience specifically among pharmacy students in Saudi Arabia are limited (8, 30). This study aimed to fill this gap by (1) assessing mindfulness and resilience levels among undergraduate pharmacy students in terms of total and sub-scale score and among the different study groups (2), explore the relationship between manfulness and resilience levels in terms of total and sub-scale scores (3), explore the relationship between mindfulness (total and sub-scales) and resilience scores and student GPA.

It is worth noting that earlier research studies that investigated mindfulness and resilience have utilized variety of research scales and tools. For example, a noticeable amount of research has used the FFMQ (31–34), while others have utilised the short form of FFMQ which included a total of 15 items (35, 36). However, resilince was measured using the BRS in the majority of the previous studies (31, 32, 34). The Conner and Davidson resilience scale was utilized in Zahra & Riaz (35) research. Also, it is important to note that prior research that utilized FFMQ and BRS have presented either the toatal score or the average score which is calculated by dividing the total score by the total number of items in the scale.

## Methods

### Study design and study period

The study used a cross-sectional design and self-administered online questionnaires. It was conducted between May and August 2023.

### Setting

The study was conducted at the College of pharmacy, King Khalid University, in the Southern region of Saudi Arabia.

### Participants

The study population was final year undergraduate pharmacy students, Year 5. Inclusion criteria were as follow: Year 5 undergraduate pharmacy students aged 18 years or older. Exclusion criteria were as follow: students enrolled in undergraduate programs other than Pharmacy, students below the age of 18 and pharmacy students in year 1 to 4.

### Sample size and sampling procedure

A non-probability convenient sample was selected. An invitation was sent to students enrolled in Pharmacy Management Course through the online management system (Blackboard). Reminders were sent at 2, 4 weeks intervals through Blackboard. Choosing final year students was because they have probably encountered various university life challenges during their five-year Pharmacy program. Participation in the study was voluntary, but students were encouraged to take part in the study. Recruitments were through Blackboard announcements and direct in-person recruitment in classes. The sample size was determined based on the total number of students enrolled in the course (*n* = 131) and determined using Roasoft sample size calculator (http://www.raosoft.com/samplesize.html; Accessed on 14 May 30, 2023) with a predetermined margarine of error of 5% and a confidence level of 95%. In order to minimize erroneous findings and increase study reliability, the target sample size was set at 98 students.

#### Inclusion criteria

To be eligible for this study, participants must be final-year pharmacy students who are 18 years of age or older. Additionally, they must be willing to voluntarily participate in the research.

#### Exclusion criteria

Participants were excluded from the study if they were pharmacy students from institutions other than the one conducting the research, or if they were in their first through fourth year of the pharmacy program. Additionally, students who declined to participate in the study were also excluded.

### Data collection tool

The survey contained three sections. Section one collected demographic data including age, gender, GPA, previous diagnosis with mental illness. Section two used the Five Facet Mindfulness Questionnaire (FFMQ) which is a validated tool used to assess mindfulness (37). FFMQ consists of 39 items distributed in five domains, observing (8 items), describing (8 items), acting with awareness (8 items), non-judging, of inner experience (8 items), nonreactivity to inner experience (7 items). It uses a five 5-point Likert scale (Never or rarely true, rarely true, sometimes true, often true, very often or always true). The minimum score in FFMQ is 39 points, while the maximum possible score is 195 points, which reflects the highest level of mindfulness. Section three used the Brief Resilience Scale (BRS), Which is a validated questionnaire to assess resilience (38). It consists of 6 items and uses a 5-point Likert Scale (Strongly disagree, disagree, neutral, agree, strongly agree). BRS has a minimum possible score of 6 points and a maximum possible score of 30 points which corresponds with highest level of resilience (38). The average scores of resilience and mindfulness are calculated by dividing the total score by the total number of items, i.e., 6 and 39, respectively.

The total score on the general scale of the FFMQ is calculated by summing the scores of all 39 items, which range from 39 to 195. It is important to note that some items are scored in reverse (items 3, 5, 8, 10, 12, 13, 14, 16, 17, 18, 22, 23, 25, 28, 30, 34, 35, 38, 39). Each subscale score ranges from 8 to 40, with the exception of the “nonreactivity to inner experience” dimension, which ranges from 7 to 35. Higher scores across all scales indicate a greater capacity for mindfulness (37).

The BRS is a concise self-report measure designed to assess resilience, defined as the ability to recover or bounce back from stress and adversity. The scale includes six items, with three positively worded (e.g., “I tend to bounce back quickly after hard times”) and three negatively worded (e.g., “I have a hard time making it through stressful events”). Participants rate each item on a 5-point Likert scale, ranging from 1 (strongly disagree) to 5 (strongly agree). Negatively worded items are reverse-scored (items 2, 4, 6). The overall BRS score is calculated as the mean of the six items, with higher scores reflecting greater resilience (39).

### Ethics approval

An ethical clearance was given by the Ethics Committee at King Khalid University ECM#2023-2011. Participant information sheet that involved detailed information about the conduct of the study was presented on the first page of the questionnaire. Written informed consent was obtained from all participants. Participation in the study was completely voluntary, and students had the right to decline the invitation without any consequences. Data was securely kept by the study investigators.

### Statistical analysis

SPSS version 28.0 for Mac was used to analyze the collected data. Descriptive statistics were used to summarize demographic information of the study participants, and the scores for FFMQ and BRS. Independent samples *t*-test was used to explore differences in mindfulness and resilience scores and mindfulness constructs among the different groups of students, i.e., according to gender and previous diagnosis of mental health issues. Pearson’s correlation test was used to examine the relationship between mindfulness score and student GPA; resilience score and student GPA; resilience and mindfulness scores or constructs among the students. The level of statistical significance was set at an α level of 0.05 for all analyses.

Reliability (internal consistency) of the FFMQ, FFMQ Sub-scales and BRS in the current study sample were assessed using Cronbach’s alpha coefficient. Acceptable Cronbach’s alpha values range from 0.60 to 0.70, and alpha values of 0.8 or greater indicates very good level of reliability (40).

## Results

A total of 131 students agreed to participate in the study and completed the questionnaire. Fifty-two (39.7%) of the participants were male and 79 (60.3%) were female. Twenty-three (17.6%) of them indicated that they have previously been diagnosed with a mental health problem. Full demographic data of the study sample is listed in Table 1.

**Table 1:** Demographic information of the participants (*n* = 131).

Characteristics	*n* (%)
Mean age (SD)	
23.03 (1.07)	131 (100)
Gender	
Male	52 (39.7)
Female	79 (60.3)
GPA	
5 to 4.75	39 (29.8)
<4.75 to 4.5	34 (26)
<4.5 to 4	32 (24.4)
<4 to 3.5	22 (16.8)
<3.5 to 3	3 (2.3)
<3	1 (0.8)
Previous diagnosis of mental health problem	
Yes	23 (17.6)
No	108 (82.4)

The reliability (internal consistency) of FFMQ, FFMQ Sub-scales and BRS in the current study sample were indicated in Table 2. Acceptable Cronbach’s alpha value (0.6–0.7) was evident for the BRS and one of the FFMQ subscales, i.e., describing. Very good level of reliability was noted for the FFMQ and its other subscales, i.e., observing, acting with awareness, non-judging of inner experience, and non-reactivity to inner experience.

**Table 2:** Internal consistency of FFMQ, FFMQ sub-scales and BRS.

Scale (subscale)	N of items	Cronbach’s alpha
FFMQ	39	0.77
FFMQ (Observing)	8	0.84
FFMQ (Describing)	8	0.70
FFMQ (Acting with awareness)	8	0.87
FFMQ (Non-judging of inner experience)	8	0.87
FFMQ (Non-reactivity to inner experience)	7	0.76
BRS	6	0.60

Assessing mindfulness and resilience levels among undergraduate pharmacy students, in terms of total and sub-scale scores, as well as different study groups, was one of the primary aims of this study. The findings from the mindfulness and resilience scale revealed a mean score of 116.93 (14.17) and 17.98 (3.88) respectively. Calculating the average score for both scales was found to be 3.00 (SD = 0.36) for mindfulness, and 3.00 (SD = 0.65) for resilience. No statistically significant difference was observed between male and female students, and those who had a previous diagnosis of mental health issues and those who did not in terms of mindfulness scores (Table 3). However, assessing resilience across the different study groups showed a statistically significant higher level of resilience among students who have never been diagnosed with mental health issues (M = 18.38, SD = 3.7) compared to those who reported a previous diagnosis (M = 16.13, SD =4.21), t (129) = −2.579, *p* = 0.011.

**Table 3:** Sample descriptive using independent samples *t*-test for equality of means (*n* = 131).

Sample	Total mindfulness score^*^	Total resilience score^*^
	M (SD)	M (SD)
Entire sample	116.93 (14.17)	17.98 (3.88)
Gender		
Male (*n* = 52)	116.96 (13.33)	17.67 (3)
Female (*n* = 79)	116.91 (14.78)	18.19 (4.37)
*t*-test	t (129) = 0.02, *p* = 0.984	t (129) = −0.802, *p* = 0.424
Previous diagnosis of mental health problem		
Yes (*n* = 23)	115.52 (17.28)	16.13 (4.21)
No (*n* = 108)	117.23 (13.49)	18.38 (3.7)
*t*-test	t (129) = 0.524, *p* = 0.601	t (129) = −2.579, P = 0.011

* Average score is calculated by dividing the total score by the total number of items in the scale.

Female students scored significantly higher in observing (M = 24.23, SD = 6.42) than male students (M = 21.83, SD = 6.94), t (129) = −2.028, *p* = 0.045. Conversely, male students scored significantly higher in acting with awareness (M = 27.33, SD = 6.88) than female students (M = 25.10, SD = 6.53), t (129) = 1.869, *p* = 0.032. However, statistically significant higher rating of acting with awareness was observed among the students who have never been diagnosed with mental health issues (M = 26.45, SD = 6.75) compared to those who reported a previous diagnosis with mental health issues (M = 23.78, SD = 6.35), t (129) = 1.74, *p* = 0.042. No statistically significant difference was observed between male and female students and, and those who had a previous diagnosis of mental health issues and those who did not in terms of other mindfulness constructs (Table 4).

**Table 4:** Differences in mindfulness constructs among the different groups of students using independent samples *t*-test for equality of means (*n* = 131).

Mindfulness constructs	Gender		Mental health issues	
	Male (*n* = 52)	Female (*n* = 79)	Yes (*n* = 23)	No (*n* = 108)
Observing	21.83 (6.94)	24.23 (6.42)	25.26 (6.98)	22.85 (6.60)
	t (129) = −2.028, *p* = 0.045		t (129) = −1.537, *p* = 0.118	
Describing	24.52 (4.90)	24.70 (5.10)	25.04 (4.90)	24.54 (5.04)
	t (129) = −0.197, *p* = 0.844		t (129) = −0.440, *p* = 0.661	
Acting with Awareness	27.33 (6.88)	25.10 (6.53)	23.78 (6.35)	26.45 (6.75)
	t (129) = 1.869, *p* = 0.032		t (129) = 1.74, *p* = 0.042	
Nonjudging of inner experience	25.46 (7.31)	23.84 (6.22)	22.35 (5.76)	24.94 (6.82)
	t (129) = 1.364, *p* = 0.175		t (129) = 1.69, *p* = 0.093	
Nonreactivity to inner experience	17.83 (4.6)	19.05 (4.97)	19.09 (4.95)	18.45 (4.84)
	t (129) = −1.419, *p* = 0.158		t (129) = −0.567, *p* = 0.571	

This study also aimed to explore the relationship between manfulness and resilience levels, in terms of total and sub-scale scores. Findings showed significant positive association between resilience and mindfulness scores of the students (r = 0.45, *p* < 0.001). Assessing the correlation between resilience and different mindfulness constructs showed a significant positive correlation between resilience and describing and acting with awareness mindfulness. Weak positive corelation was observed between resilience and all other mindfulness constructs, i.e., observing, nonjudging of inner experience and nonreactivity to inner experience (Table 5).

**Table 5:** Correlation between resilience and mindfulness constructs (*n* = 131).

Mindfulness constructs	Resilience	
	Pearson correlation	*p*-value
Observing	0.06	0.497
Describing	0.387	<0.001
Acting with Awareness	0.318	<0.001
Nonjudging of inner experience	0.163	0.063
Nonreactivity to inner experience	0.167	0.057

Another aim was to explore the relationship between mindfulness (total and sub-scales) and resilience scores and student GPA. There was a weak positive association found between student GPA and mindfulness score (r = 0.128, *p* = 0.147). Likewise, there was a weak positive association observed between student GPA and the four FFMQ subscales observing, describing, non-judging of inner experience and non-reactivity to inner experience (Table 6). Assessing the association between student GPA and resilience score showed a weak association (r = −0.097, *p* = 0.273).

**Table 6:** Correlation between student GPA and mindfulness constructs (*n* = 131).

Mindfulness constructs	GPA	
	Pearson correlation	*p*-value
Observing	0.079	0.370
Describing	0.089	0.315
Acting with Awareness	−0.018	0.838
Nonjudging of inner experience	0.040	0.652
Nonreactivity to inner experience	0.142	0.105

## Discussion

Although factors such as resilience and mindfulness that are recognized to be positively associated with individual’s mental health and psychological well-being, there remains a notable paucity of research in this area especially in healthcare academic programs like pharmacy (8, 20, 30). Therefore, it is crucial to investigate mindfulness and resilience among undergraduate students. The current research was set out with the aim of evaluating mindfulness and resilience levels among undergraduate pharmacy students in a public institution in the South-west of Saudi Arabia. It further aimed to examine the relationship between mindfulness and resilience as well as evaluating the association between mindfulness and resilience, and student GPA.

Findings from the current research revealed an average mindfulness (3.00, SD = 0.36) and an average resilience scores (3.00, SD = 0.65) that are comparable to what was reported in the previous international and national research using the FFMQ and the BRS with score difference that was within ±5% from each other, and both reflect intermediate levels of mindfulness and resilience (31–33). Specifically, in the UK, university students enrolled in different undergraduate and postgraduate degrees across five primary subject areas (arts and humanities, engineering and technology, life sciences and medicine, natural sciences, and social sciences and management) scored on average 3.14 (SD = 0.57) in mindfulness and 3.10 (SD = 0.91) in resilience (31). Similarly, an Australian study that assessed resilience level among veterinary students in their clinical years of study showed an average score of 3.16 (SD = 0.82) (32). Additionally, the reported mindfulness score in the study of Alzahrani et al. (33) that targeted medical students in a Saudi university was 3.05 (SD = 0.4), which is almost identiacl to our study sample with only 1.67% diffrence noted (33). In contrast to our study findings, higher levels (with >5% difference) of mindfulness (3.27, SD = 0.50) and resilience (3.46, SD = 0.76) were observed in a sample of undergraduate American African students attending a historically black university (34). The disparity between the findings of this study and ours could be attributed to the impact of other encounters with hardship that may have shaped the mindfulness and resilience of African American students.

In agreement with a previous report (16), findings from the current study showed that female students scored statistically significant higher in observing mindfulness than their male counterparts. On the other hand, male students scored statistically significant higher in acting with awareness mindfulness than females. This could be attributed to the gender characteristics difference, particularly the cognitive functioning difference that exists between the two genders (16). Females are believed to be better than males in handling multiple tasks at the same time, however, males tend to focus on one task at a time while being aware of it (41).

In fact, results from previous research on gender-specific differences of mindfulness varied across studies (42). Some studies concluded that mindfulness was not associated with gender, while others indicated statistically significant differences (42). For example, a Greek study that evaluated mindfulness among school and university students showed that females had lower mindfulness scores than males (43). Similar findings were reported in a study conducted on athletes in Japan who belonged to their university athletic clubs (44), and another study conducted on undergraduate students in the United States (45). A study assessed mindfulness among nursing staff in Italy reported overall higher mindfulness among males, but females scored higher in observing construct (46) which is in accordance with our findings.

Another important finding from the current research showed that students who had never been diagnosed with mental health issues scored significantly higher in acting with awareness compared to those who reported a previous mental health diagnosis. In support of our findings, previous research suggested a negative correlation between the acting with awareness component of mindfulness and psychological issues (47–49). These findings underscore the importance of developing tailored interventions and programs aimed at fostering the acting with awareness facet of mindfulness in students, thereby improving their tolerance to distress and protect against mental health issues (50).

.Likewise, findings from the current research showed higher levels of resilience among students who had never been diagnosed with mental health issues compared to those with a reported history. This is not surprising since psychological well-being is recognized as the most identified predictor of resilience in a recent systematic literature review (51). This highlights the importance of implementing targeted programs to foster resilience, enhance students’ ability to cope, and equip them with tools to bounce back in the face of adversity (51).

Mirroring earlier findings (31–35), the results of this study showed that mindfulness and resilience are significantly associated. Additionally, a strong positive corelation was found between describing and acting with awareness mindfulness and resilience. This suggests that individuals who had higher levels of describing and acting with awareness mindfulness also showed higher levels of resilience. Findings on the corelation between resilience and the different mindfulness dimensions were inconsistent across studies. For example, McArthur et al. (32) reported that students who scored higher in non-judgmental mindfulness, acting with awareness mindfulness, and observing mindfulness scored higher in resilience (32). Another report demonstrated that the nonreactivity facet of mindfulness was significant associated with resilience (34). Additionally, a meta-analysis that investigated the association between mindfulness and resilience among the university students showed that acting with awareness and nonreactivity to inner experience were the most significant dimensions associated with the university student resilience (3).

Investigating the corelation between mindfulness and resilience, and student GPA showed a weak association. The effect of mindfulness on academic achievement has been investigated in various previous studies (52, 53), and significant corelation was observed. Mindfulness practices have shown potential benefits on academic performance. It has been suggested that mindfulness improves attention and concentration (54). Not only this but it also enhances emotional processing and cognitive skills. Other positive outcomes include working memory enhancement, mind wandering reduction (52). These outcomes play an important role in effective learning, information processing and hence better academic achievements (55). Discrepancies between our findings and previous research findings suggest that other mediating factors such as student motivation might have influenced the strength of the relationships between the investigated variables. This could lay the foundation for further research that aim at exploring these factors.

### Study limitations

Several limitations to the current study need to be acknowledged. First, the study was conducted at a single pharmacy institution targeting final year undergraduate students. Therefore, the study findings cannot be genialized beyond similar contexts. Large-scale research targeting different regions of the country is recommended. Second, the use of a cross-sectional design has imposed limitation to the study findings as causality cannot be established. Further research might explore the impact of mindfulness interventions on student resilience as well as their academic performance. The cross-sectional design of the study also limits its ability to determine the temporal relationship between mindfulness and the onset of mental health conditions. Future research would benefit from longitudinal designs or a more explicit consideration of the directionality of the relationship. Without such data, it remains unclear whether mindfulness acts as a protective factor or emerges as a consequence of managing mental health challenges.

## Conclusion

This study was conducted to evaluate Pharm D students’ mindfulness and resilience using the FFMQ and BRS, respectively. It also aimed to explore the relationship between mindfulness as a human capacity and resilience as well as their effects on GPA as an indication of academic performance. Higher level of observing mindfulness was reported among female students, while male counterparts reported higher level of acting with awareness. Additionally, higher level of resilience was observed among students who have never been diagnosed with mental health issues compared to those who reported a previous diagnosis. Students who had never been diagnosed with mental health issues scored significantly higher in acting with awareness. Therefore, tailored interventions and programs could be implemented to foster resilience, enhance students’ ability to cope, and equip them with tools to bounce back in the face of adversity. A strong positive corelation was found between resilience and describing as well as acting with awareness mindfulness. This suggests that individuals who had higher levels of describing and acting with awareness mindfulness also showed higher levels of resilience. Contrary to previous research, the corelation between mindfulness and resilience, and student GPA showed a weak association. Further research could explore other factors that might influence the strength of the relationships between these variables.

## Data Availability

The raw data supporting the conclusions of this article will be made available by the authors, without undue reservation.
